# Notes and News

**Published:** 1906-02-10

**Authors:** 


### Notes and News.

H.R.H. The Princess Royal has graciously consented to lay the foundation-stone of the new wing now being built at the Bolingbroke Hospital, Wandsworth Common. Her Royal Highness will be accompanied by the Duke of Fife. The date, which will probably be at the end of April, will be announced as soon as it has been fixed by Her Royal Highness.

There was a large gathering of women sanitary inspectors last Saturday afternoon at the Cavendish Rooms, Mortimer Street, when the President of their society was "at home" to the members and friends. The guests included Sir James Crichton Browne, Sir Shirley Murphy, Dr. King Warry, and Dr. Collingridge.

There is said to be poison in the flower stalk of one of the popular spring flowers—the daffodil. Complaints on the part of men working in the nurseries of a large daffodil grower led to inquiries which have resulted in the discovery that the juice of the plant contains crystals of lime, which, if the hands are cut or chapped, set up violent irritation.

An outbreak of typhoid fever is reported on board the Swedish training smp *Frej a*, which is now at Constantinople. Prince William of Sweden who is on board the vessel, will in consequence of the epidemic, be compelled to prolong his stay in the Turkish capital. In accordance with the Sultan's orders the ship has been thoroughly disinfected and drinking water is supplied to the men at his personal expense.

A remarkable mistake in burial occurred at Liverpool this week. Two infants belonging to different parents were taken from different houses to a fever hospital in the city. One died and was buried, and the other on its recovery was handed to its supposed mother. The latter, however, declared that the child was not hers, and inquiries showed that it belonged to the other woman who had believed that her baby was dead.

A number of cases of ptomaine poisoning have occurred at Accrington which are attributed to the consumption of potted meat. In several instances whole families have suffered.

In the annual report just issued by the Inspector to the Chorlton Union under the Infant Life protection Act of 1897, it is strongly urged that the Act should be strengthened by the inclusion within its observation of women who take only one child to nurse, and by new legislation to deal with cases where children are adopted in consideration of the payment of a lump sum. Eight years' experience has confirmed the conviction of the Inspector that every child maintained for hire or received apart from its parents should be protected by the Act.

The Indian Government, in the report which they have just issued, embodying the conclusions drawn from the experience gained during the last five years in endeavouring to check the spread of plague, indicate among the preventive measures which they consider essential, the destruction of rats. The report says that the most conspicuous change in the opinion of experts in India regarding plague since July 1900, is the greatly increased importance now ascribed to the part played by rats in spreading and keeping alive the disease. The Government consider it as necessary to the safety of the community to destroy infected rats as to segregate plague-stricken people; and, in fact, they are of opinion that "almost all evidence regarding the causation of plague may be regarded as pointing to the rat as the chief agent in its diffusion."

As the result of a personal investigation an American has recently brought to light some interesting facts in connection with the quack medicine industry. Those who write to the quack-medicine vendor and confide in him the nature of their troubles, doubtless believe that their letters are read by a skilled physician, that he gives each case his careful consideration—which from the sheer incoherence of the letters is often an impossibility—and then either destroys the letters or files tnem for future reference. As a matter of fact what frequently does happen is this: The letters, having served their original purpose, are sold to "letter-brokers" at something like a farthing each, and are bought by other patent-medicine dealers, who like to get the addresses of persons who are likely customers. Among the letters which the individual referred to found for sale were "55,000 female complaint letters, 44,000 bust developer letters, 40,000 women's regulator letters, 7,000 paralysis letters, 9,000 narcotic letters, 52,000 consumption letters, 3,000 cancer letters, and 65,000 deaf letters." Besides these there were for sale nearly a hundred thousand letters dealing with other complaints! And these, received as confidential statements, were, having served to sell one nostrum, offered to the vendor of the next in order that he might have a ready-made *clientèle* to address. Here we have a confessional which sells its secrets at a farthing apiece. What is to be said of it?

